# Novel insights into *Radix Pseudostellariae* saponins assist *Mycoplasma gallisepticum* attenuated vaccine to enhance the immunoprotection of chicken

**DOI:** 10.1128/msystems.00781-26

**Published:** 2026-08-31

**Authors:** Qixian Feng, Lihua Liao, Yihua Gong, Jingru Xu, Xiangduan Wei, Zhengrun Xiao, Pengqiang Chen, Beilei Chen, Kaizhao Zhang, Quanxi Wang

**Affiliations:** 1 Fujian Key Laboratory of Traditional Chinese Veterinary Medicine and Animal Health/University Key Laboratory for Integrated Chinese Traditional and Western Veterinary Medicine and Animal Healthcare in Fujian Province, College of Animal Science, Fujian Agriculture and Forestry University 12449, Fuzhou, China; Pacific Northwest National Laboratory, Richland, Washington, USA

**Keywords:** *Radix Pseudostellariae *saponins, *Mycoplasma gallisepticum-*attenuated vaccine, mucosal immunity, *Lactobacillus* sp. UMNPBX13, phosphatidylcholine

## Abstract

*Mycoplasma gallisepticum* (MG) is a major pathogen causing chronic respiratory disease in chickens, and the MG-attenuated vaccine (MGAV) often fails to induce effective mucosal immunity. *Radix Pseudostellariae* saponins (RPS), important bioactive components of the traditional Chinese herb, have been utilized for immunomodulation, although the underlying mechanisms remain to be elucidated. In this study, chickens were randomly assigned to groups receiving intranasal MGAV alone, MGAV plus RPS at different doses, or appropriate controls. Serum, tracheal mucosa, and respiratory lavage samples were collected for antibody measurement, cytokine profiling, histological assessment, and metagenomic/metabolomic analyses. RPS significantly increased MG-specific antibody levels and enhanced expression of the immunomodulatory cytokines, while suppressing that of pro-inflammatory factors. It also promoted tracheal mucosal integrity by elevating goblet cell numbers and mucosal epithelial height. Through metagenomic and metabolomic analysis, RPS was found to enrich the respiratory bacterium *Lactobacillus* sp. UMNPBX13, which was positively correlated with the metabolite phosphatidylcholine (PC). Intranasal administration of *Lactobacillus* increased mucin 2 (*MUC2*) and PC levels in tracheal mucosa. Similarly, PC combined with MGAV enhanced mucosal immunity by modulating cytokines and increasing MG-specific antibodies. These findings demonstrate that RPS enhances MGAV-induced respiratory mucosal immunity and provides a novel adjuvant strategy for improving poultry respiratory disease vaccines by modulating the respiratory microbiota, specifically through enrichment of *Lactobacillus* sp. UMNPBX13, which promotes PC production.

**IMPORTANCE:**

*Mycoplasma gallisepticum* (MG) constitutes a notable challenge to poultry health. Although the MG-attenuated vaccine (MGAV) is widely used for disease control, it often fails to induce robust mucosal immunity. Here, we demonstrate that intranasal co-administration of *Radix Pseudostellariae* saponins (RPS) with MGAV augments mucosal immunoprotection. Integrated metagenomic and metabolomic analyses revealed that RPS enriches respiratory *Lactobacillus*, which in turn promotes the production of its key metabolite phosphatidylcholine (PC), thereby supporting mucosal immune enhancement. These findings offer a promising strategy for improving poultry vaccine efficacy and may provide a basis for future exploration of mucosal vaccination approaches in other animal species.

## INTRODUCTION

Chronic respiratory disease (CRD) induced by *Mycoplasma gallisepticum* (MG) is a widespread infectious condition in global poultry production systems (1). This disease is characterized by mucous catarrhal inflammation and severe air sacculitis in chickens, leading to substantial economic losses for poultry producers across diverse geographic regions (2). A critical initial stage in MG infection and pathogenesis involves pathogen adhesion to host cells, which typically impairs the tracheal mucosa or causes ciliary loss, leading to CRD (3). As a non-invasive bacterium, MG firmly adheres to the respiratory epithelial surface, thereby evading clearance mechanisms such as mucociliary escalator function and phagocytic uptake (4). The subsequent accumulation of mucus and pathogens precipitates inflammatory lesions in the trachea and air sacs, adversely affecting respiratory health (5). Consequently, the immunocompetence of the respiratory tract, particularly the trachea, constitutes a key determinant in controlling MG infection. Currently, vaccination serves as the primary measure to prevent MG outbreaks (6). Commonly used commercial live-attenuated MG vaccines include the F and Ts-11 strains (7). Although the protective effect of such vaccines relies on memory B cells in the trachea, MG-specific antibodies might also be produced locally within the tracheal mucosa (8). Despite being attenuated, these vaccine strains retain residual pathogenicity and may still elicit post-vaccination reactions, including localized inflammatory responses (9). Respiratory tract infections can modify the immune microenvironment within the mucosa (10). Compared to traditional intramuscular vaccines, intranasal vaccines induce mucosal immunity in the respiratory tract and even in distal mucosal tissues (11). Effective mucosal immunity is recognized as a key factor accounting for the difference between mild and severe patients, aiding in the control of pathogens in the upper respiratory tract (12). One study reported that a nasally delivered live-attenuated influenza vaccine, designed with chimeric antigens targeting influenza B virus (IBV), elicited broad systemic and mucosal immune responses, conferring robust cross-lineage protection against diverse IBV strains (13). In another study, transcriptomic sequencing of tracheal tissues from chickens that achieved 100% protection against infectious bronchitis virus challenge revealed upregulation of B-cell receptor signaling and enrichment of immunoglobulin A (IgA) production pathways, demonstrating that secretory IgA (sIgA)-mediated mucosal immunity serves as the key protective mechanism (14).

Plant saponins, natural compounds extracted from seeds, roots, stems, and leaves of various plants, exhibit multiple biological activities, including immunomodulatory, anti-inflammatory, and hypoglycemic effects (15). The field of pharmacognosy has long recognized the importance of natural compounds in drug discovery and development. Among them, *Radix Pseudostellariae*, the dried tuberous root of *Pseudostellaria heterophylla*, has attracted considerable attention as a source of diverse chemical constituents, including polysaccharides, cyclic peptides, saponins, amino acids, and other bioactive compounds (16). These components exhibit diverse pharmacological activities, such as modulation of blood glucose levels, antitumor effects, attenuation of inflammatory responses, and enhancement of antioxidant capacity. The broad therapeutic potential of *Pseudostellaria heterophylla* underscores its relevance in the search for novel therapeutic agents (17). Notably, saponins have garnered interest as vaccine adjuvants because of their ability to modulate immune cells (e.g., T cells) and activate antigen-presenting cells (APCs). They can promote either pro-inflammatory helper T cell (Th)1/Th2 responses or induce anti-inflammatory Th2 immunity, critically shaping the quality and magnitude of adaptive immune responses and influencing vaccine efficacy (18). For instance, saponin-based adjuvants such as AS01 and QS-21 have been shown to activate APCs, with their intramuscular injection triggering the release of inflammatory cytokines in draining lymph nodes to enhance immune activation (19). Furthermore, a study showed that severe acute respiratory syndrome coronavirus 2 (SARS-CoV-2) S1-Fc fusion protein-based candidate vaccines formulated with a saponin microemulsion adjuvant induced high titers of S1-specific neutralizing antibodies in cynomolgus monkeys (20). Beyond their adjuvant properties, plant-derived compounds have also demonstrated direct therapeutic effects on respiratory diseases. In a separate study, phillygenin, a bioactive compound from *Forsythia suspensa*, was demonstrated to alleviate IBV-induced inflammation by reducing viral loads and pro-inflammatory cytokines (interleukin-6 [IL-6], tumor necrosis factor-α [TNF-α], and IL-1β) primarily through inhibition of the TLR7/MyD88/NF-κB pathway. Interestingly, phillygenin-treated broilers exhibited a higher abundance of *Lactobacillus* in the respiratory tract, indicating that certain plant-derived compounds may positively modulate the respiratory microbiota while exerting antiviral effects (21). Similarly, aerosol inhalation of total ginsenosides (TG) from Panax ginseng has been reported to significantly attenuate acute lung injury and pulmonary fibrosis in a mouse model, as evidenced by the regulation of interferon-γ (IFN-γ), IL-1β, and transforming growth factor-β1 (TGF-β1) levels, reduced inflammatory cell infiltration, and restoration of alveolar architecture (22). The adjuvant potential of saponins is further evidenced by their capacity to elicit robust mucosal and systemic immune responses following intranasal administration, including immune activation within the respiratory mucosa, nasal-associated lymphoid tissue, and cervical lymph nodes (23).

Despite these advancements, research on the application and immunomodulatory effects of *Radix Pseudostellariae* saponins (RPS) co-administered with attenuated MG vaccine (MGAV) remains limited. This study aimed to define the mechanism by which RPS enhances the mucosal barrier integrity. Analysis of the respiratory microbiota and metabolome identified differential bacteria and metabolites through which RPS enhances the mucosal barrier, thereby clarifying its protective role against mycoplasma infection. Analysis of the respiratory microbiota and metabolome identified differential bacteria and metabolites through which RPS enhances mucosal barrier, thereby clarifying its protective role against mycoplasma infection.

## MATERIALS AND METHODS

### Animals and drugs

A total of 90 healthy 30-day-old Hetian chickens (45 male and 45 female), with an average body weight of 546 ± 15 g were purchased from the Hetian Chicken Conservation Farm in Changting County, Longyan City, Fujian Province, China. All birds were confirmed negative for MG antigen by polymerase chain reaction (PCR) and for MG antibody by enzyme-linked immunosorbent assay (ELISA) prior to the experiment. The flock had received the CVI988 liquid nitrogen vaccine for Marek’s disease and an inactivated bivalent oil-emulsion vaccine against Newcastle disease and avian influenza. After a 3-day acclimatization period, the experiment commenced.

RPS (80% purity) was purchased from Lanzhou Wotelaisi Biological Technology Co., Ltd. RPS content was quantified using the vanillin–sulfuric acid colorimetric method, with Ginsenoside-Re (Shanghai Yuanye Bio-Technology Co., Ltd, B21055) as the reference standard. The standard curve was generated by plotting the absorbance at 560 nm (*y*-axis) against the concentration of Ginsenoside-Re (*x*-axis) over the range of 0.2–1.2 mg/mL, yielding the equation *y* = 2.7197*x* + 0.1036 with a correlation coefficient of *R*^2^ = 0.999. Using this calibration, two independent RPS samples weighing 1.10 and 1.20 mg were analyzed, giving total saponin contents of 73.95% and 72.42%, respectively. The average total saponin content of the RPS preparation was thus determined to be 73.18%.

### Animal grouping and immunization

Chickens were randomly allocated into five groups (*n* = 18/group): control (0.1 mL saline), MGAV (3.0 × 10^6^ color-changing units [CCUs]/bird), H-RPS (MGAV + 100 mg/kg RPS), M-RPS (MGAV + 20 mg/kg RPS), and L-RPS (MGAV + 5 mg/kg RPS). All groups received intranasal administration at a volume of 0.1 mL per bird. The primary immunization was followed by a booster at day 28. Serum and tracheal mucus samples were collected from live chickens prior to primary immunization, and at 3 days, 1, 2, 3, and 4 weeks post-primary immunization, as well as at 1, 2, and 3 weeks post-secondary immunization. Tracheal tissues and spleens were also collected at each corresponding time point from euthanized chickens designated for terminal sampling. The excised trachea was fixed in 4% paraformaldehyde. Tracheal mucus was flushed with 2 mL RNA preservation solution (Servicebio) and collected together with the remaining tracheal tissue.

### ELISA

The positivity and dynamic changes in serum titers of MG-specific antibodies were determined using a commercial ELISA kit (Cat# MG-06-01144-05, IDEXX). Concentrations of interferon (IFN)-γ (Cat# ZC-51740), interleukin (IL)-4 (Cat# ZC-51662), IL-5 (Cat# ZC-51662), IL-17A (Cat# ZC-56527), IL-6 (Cat# ZC-51663), IL-1α (Cat# ZC-56351), and C-C chemokine ligand 4 (CCL4; Cat# ZC-56549) were measured using kits from ZCIBIO Technology Co., Ltd. The concentrations of phosphatidylcholine (PC) (Cat# YX-160300C, Shanghai Yuanxin Biotechnology Co., Ltd.), total sIgA (Cat# ZC-51833, ZCIBIO), and mucoprotein 2 (MUC2; Cat# ml060917, Shanghai Enzyme-linked Biotechnology Co., Ltd.) in tracheal tissue and mucus were assessed using the corresponding commercial kits, as specified.

### RNA extraction and reverse transcription quantitative real-time (RT-qPCR) analysis

Total RNA was isolated from samples using the TransZol Up Plus RNA Kit (Cat# ER501, TransGen), and cDNA was synthesized using the NovoScript Plus All-in-one 1st Strand cDNA Synthesis SuperMix with gDNA Purge (Cat# E047-01B, Novoprotein), as previously described (24). Primer sequences are listed in Table 1.

**TABLE 1:** Primer sequences

Gene name	Sequences (5′−3′)	Size (bp)
MG-F	AGGTAAACCTGGTTACGGGAAAAGC	214
MG-R	GATCTGAACGTCGTCGATCATTAACA	
IFN-γ-F	GTGAAGAAGGTGAAAGATATCATGGA	125
IFN-γ-R	GCTTTGCGCTGGATTCTCA	
IL-4-F	CTCACTGCCCACCCTGCTG	129
IL-4-R	ACCTCTCCCTGGATGTCATTCAC	
IL-1β-F	AACATCGCCACCTACAAG	84
IL-1β-R	GACGGTAATGAAACATAAACG	
MUC2-F	CTCTTACCACCATAGTTACCACAAGTC	128
MUC2-R	CATAGTCACCACCATCT TCTTCAGG	
β-Actin-F	GGCGGTTTGCGGGTTGAC	120
β-Actin-R	CATCACCAGAGTCCATCACAAT	
*Lactobacillus_sp._UMNPBX13*-F	TTATGCCTCGCCAAATCGCTATG	102
*Lactobacillus_sp._UMNPBX13*-R	AAACGGTGGTATGGTCTTTGCC	
*Ruminococcuss_sp._OF05-2BH*-F	TTTCAAGGCTTCTTCCCATTCATTCC	108
*Ruminococcuss_sp._OF05-2BH*-R	TGCGTATTGGCTGGATGATTATAGTC	
*Prevotella_sp._P5-108*-F	GGCGGTTTGCGGGTTGAC	98
*Prevotella_sp._P5-108*-R	AGCGGCGACAGCGACAG	
16S rRNA-F	AGAGTTTGATCCTGGCTC	133
16S rRNA-R	TGCTGCCTCCCGTAGGAGT	

### Flow cytometry

Spleens were collected from chickens at 1 and 2 weeks (*n* = 3 per time point) post-primary immunization. A splenic lymphocyte suspension was prepared in accordance with our previously described method (25). Briefly, the cell suspension was incubated with the following fluorescence-conjugated mouse anti-chicken monoclonal antibodies in the dark for 30 min: anti-chicken CD3-APC (Cat# 8200-11, SouthernBiotech), CD4-PE (Cat# GTX43093-100, GeneTex), and CD8α-FITC (Cat# GTX43479, GeneTex). The proportions and counts of CD3^+^, CD4^+^, and CD8^+^ T lymphocytes were subsequently analyzed using a NovoCyte 2000 flow cytometer (Agilent).

### Histopathology analysis

Tracheal tissues were excised and fixed in 4% paraformaldehyde. For histological examination of the tracheal mucosa and goblet cells, tissue sections were stained with Alcian blue (AB) and periodic acid-Schiff (PAS) to visualize acidic and neutral mucins, respectively. The height of the tracheal mucosal epithelium and the area of goblet cells were measured on PAS-stained sections. Three random fields of view were selected and quantified on both AB- and PAS-stained sections using Image-Pro Plus 6.0 software.

### Challenge protection assay and PCR-based pathogen detection

Chickens in the control, MGAV, and three MGAV + RPS groups were challenged with the pathogen, alongside a healthy group (*n* = 6) that remained unchallenged. Each challenged bird received intranasal inoculations with 10^9^ CCU of the MG mx-FZ2023 strain on three consecutive days. Tracheal and lung tissues were collected at 2 weeks post-challenge. Tissues were embedded in paraffin and subjected to hematoxylin and eosin (H&E) staining for pathological examination. Total DNA was extracted from lung tissues using the TransZol Up Plus DNA Kit (Cat# ER501, TransGen), and PCR amplification was performed to detect MG genomic material, as previously described (26).

### Lung lesion scoring

Lung lesions in chickens were scored following a reference scoring method for slaughterhouse pneumonic diseases (27). Chicken lungs are spongy and unlobed, with dorsal surfaces embedded between the ribs; therefore, scoring was performed on each lung as a whole. The extent of consolidation in each lung was scored on a scale of 0–4, with a maximum score of 4 per lung and 8 per bird. Lesion severity was calculated as follows: percentage of MG-induced lung lesion area = (sum of consolidated areas on the dorsal and ventral surfaces/total lung area) × 100%. Morbidity and mortality rates were calculated as follows: (number of sick or dead chickens/total number of challenged chickens) × 100%. The total lung lesion score for each group was obtained by summing the individual scores of all chickens within the group (Table 2).

**TABLE 2:** The scoring rules of lung lesions in chickens

Lung lesion area	Score
No lesions in the lungs	0
Lesion area ≤ 25% (slight)	1
26% ≤ lesion area ≤ 50% (moderate)	2
51% ≤ lesion area ≤ 75% (severe)	3
Lesion area > 75% (extremely severe)	4

### Metagenomic analysis

Metagenomic analysis was performed on tracheal mucus samples. Raw sequencing data were quality-controlled using fastp (v0.23.2, https://github.com/OpenGene/fastp), and read counts were summarized at each step. Quality-controlled reads were then aligned to the host genome using BBMap for removal of host-derived sequences. Filtered reads were assembled *de novo* using MEGAHIT (v1.1.2, https://github.com/voutcn/megahit), which constructs a De-Bruijn graph based on k-mer overlaps to generate contigs; those >500 bp were retained for downstream analysis. Open reading frames were predicted from the assembled contigs, and a non-redundant gene catalog was constructed for functional annotation. Taxonomic classification was performed by aligning gene sequences against the NCBI NR database, using annotation parameters (28). To validate the metagenomic sequencing data, quantitative PCR targeting the bacterial 16S rRNA gene was performed as a reference.

### Metabolomics analysis

To investigate the effect of RPS on tracheal metabolites in chickens, liquid chromatography with tandem mass spectrometry (LC–MS/MS) was employed to acquire both primary and secondary mass spectra. Raw mass spectrometry data were processed using Progenesis QI software (v3.0) for metabolite identification. During data analysis, unsupervised principal component analysis (PCA) was first performed to assess the overall distribution of samples and the stability of the analytical process. This was followed by supervised partial least squares-discriminant analysis (PLS-DA) and orthogonal PLS-DA to distinguish overall metabolic profiles between groups and identify potential differential metabolites (29). To reliably screen for differential metabolites, a combination of multivariate and univariate analyses was applied: metabolites with a variable importance in projection (VIP) >1 in the PLS-DA or orthogonal PLS-DA models were further subjected to Student’s *t*-test, and those with a *<P* value <0.05 were considered statistically significant.

### Integrated microbiome and metabolome analysis

To systematically elucidate the mechanism by which RPS enhances immune function in chickens, Spearman correlation analysis was performed between differentially abundant bacterial species identified by metagenomics and differential metabolites identified by metabolomics, following the methodology established in our previous work (25). All metagenomic and metabolomic sequencing services were provided by OE Biotech Co., Ltd. (Shanghai, China).

### Intranasal interventions and immunomodulatory assessments in chickens

#### Intranasal *Lactobacillus* administration and MG challenge

Twelve 30-day-old healthy chicks were randomly divided into two groups (*n* = 6/group). The control group was intranasally administered 50 μL of 0.9% sterile saline, while the LAB group received 50 μL of a solution containing 1.5 × 10^9^ colony-forming units (CFUs) of *Lactobacillus reuteri* BNCC362998 (purchased from BeNa Culture Collection, BNCC, Beijing, China) for three consecutive days. On days 7, 10, and 14 post-inoculation, respiratory tract and oral mucus samples were collected using sterile cotton swabs. On day 14, three chickens from each group were randomly selected and sacrificed to collect tracheal tissues and mucus. The remaining chickens were intranasally challenged with the MG mx-FZ2023 strain (10^9^ CCUs/bird) for three consecutive days, and tracheal tissues were aseptically collected at 2 weeks post-challenge.

#### Intranasal PC effects on cytokines and tracheal MUC2 expression

Twenty-four 30-day-old healthy chickens with an average body weight of 546 ± 4.32 g were randomly assigned to four groups (*n* = 6): control group (100 μL 0.9% intranasal saline) and three treatment groups receiving 100 μL solutions containing a high (200 μg/kg), medium (40 μg/kg), or low (8 μg/kg) dose of PC. Whole blood and tracheal mucus samples were collected on post-treatment day 7.

#### PC-enhanced MGAV immunity via intranasal delivery

Eighteen 30-day-old healthy chicks, with an average body weight of 554 ± 4.15 g and confirmed negative for MG antigens and antibodies, were randomly allocated into three groups (*n* = 6/group): control (100 μL sterile saline), MGAV (100 μL vaccine), and PC + MGAV (100 μL vaccine containing 40 μg/kg PC), all administered intranasally. Serum was collected pre-administration and on post-immunization days 7, 14, and 21 for MG-specific antibody assessment. Tracheal mucus and whole blood were obtained on post-immunization days 14 and 7, respectively.

### Statistical analysis

All data used in this study were analyzed. Two-group comparisons were performed using Student’s *t*-tests or non-parametric tests, as appropriate. Multiple-group comparisons were performed using one-way ANOVA followed by Tukey’s *post hoc* test when the assumptions of normal distribution and homogeneity of variances were satisfied. All data are presented as mean ± standard error of the mean (SEM). In all bar graphs, different lowercase letters above bars indicate statistically significant differences between groups (*P* < 0.05), while groups sharing the same letter are not significantly different (*P* > 0.05). Statistical significance was defined as *P* < 0.05. All statistical analyses were performed using GraphPad Prism version 9.5.1 (GraphPad, San Diego, CA, USA).

## RESULTS

### RPS enhances MG-specific antibody production

**Fig 1:**
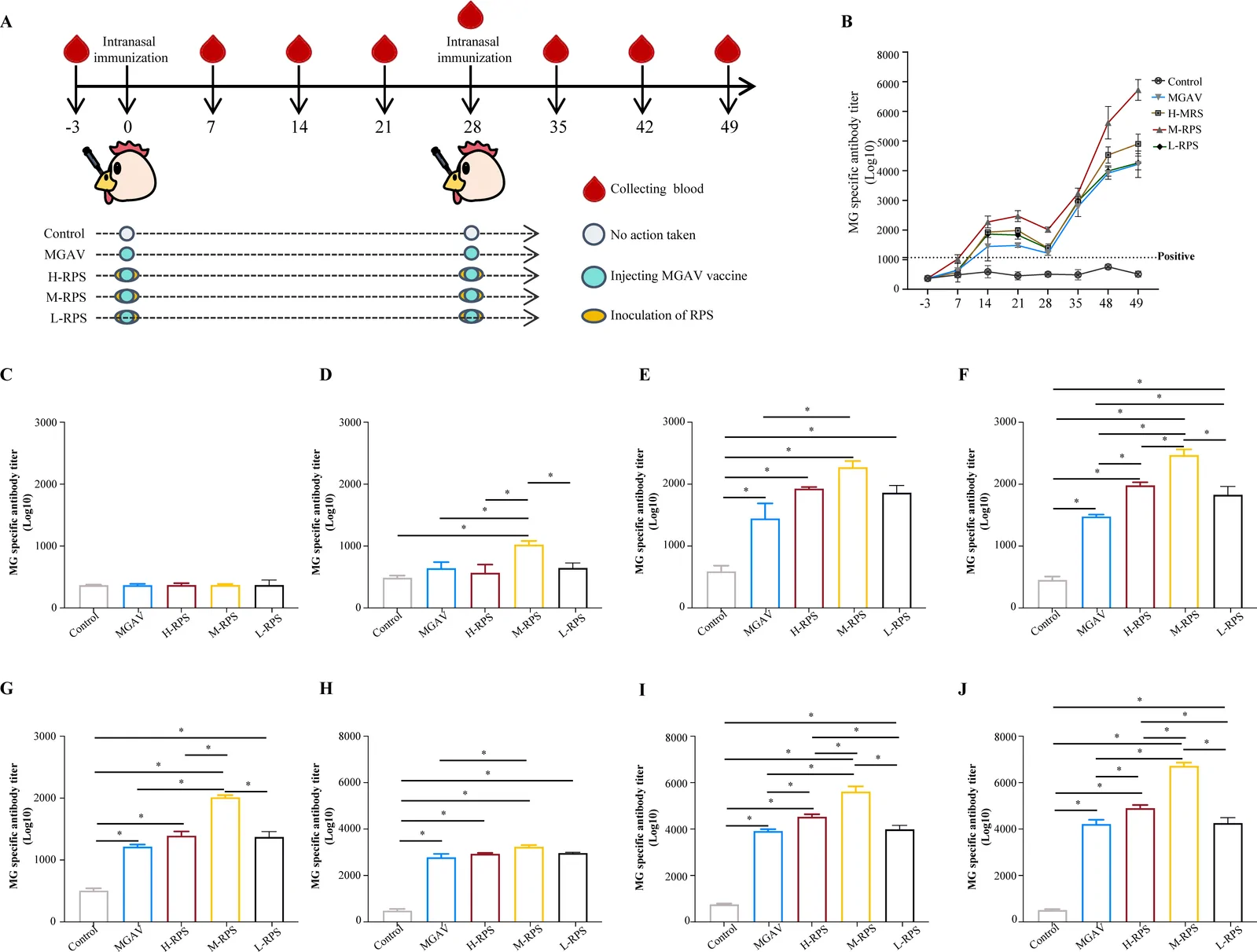
Intranasal co-administration of RPS with MGAV enhances MG-specific antibody responses. (**A**) Schematic diagram of the experimental timeline for evaluating the enhancement of MGAV-induced immune responses by RPS. (**B**) Kinetic profile of MG-specific antibody responses throughout the experimental period. (**C**) Baseline MG-specific antibody levels prior to immunization. (**D–G**) MG-specific antibody titers at 7 (**D**), 14 (**E**), 21 (**F**), and 28 (**G**) days after primary immunization. (**H –J**) MG-specific antibody titers at 35 (**H**), 42 (**I**), and 49 (**J**) days after booster immunization (chickens were intranasally immunized with MGAV alone or in combination with different doses of RPS (H-RPS: 100 mg/kg; M-RPS: 20 mg/kg; and L-RPS: 5 mg/kg). Data are presented as the mean ± SEM (*n* = 6). In panels **C–J**, statistical significance is indicated by a horizontal line with an asterisk (* for *P* < 0.05). Non-significant (*P* > 0.05) pairwise comparisons are not marked.

Intranasal co-administration of RPS with MGAV significantly enhanced MG-specific antibody titers in chickens. The experimental timeline for immunizations and blood collections is shown in Fig. 1A, and the kinetic profiles of the antibody responses are summarized in Fig. 1B. Prior to immunization, all groups showed comparable baseline antibody levels (Fig. 1C). Following primary immunization, the M-RPS group showed a significant increase in antibody titers by day 7, which was sustained and further enhanced through days 14 and 21, when both the H-RPS and M-RPS groups exhibited significantly higher titers than the MGAV group, with the M-RPS group consistently eliciting the strongest response (Fig. 1D through F). Although a general decline in antibody levels was observed by day 28, all RPS groups maintained relatively higher titers compared with the MGAV group. After the booster immunization, the M-RPS group again showed a significant antibody increase by day 7 (Fig. 1H). This effect persisted at days 14 and 21, with both the H-RPS and M-RPS groups maintaining superior antibody levels relative to the MGAV group, further confirming the robust and sustained adjuvant effect of M-RPS. The L-RPS group showed no significant improvement at most time points (Fig. 1I and J).

### Effects of intranasal co-administration of RPS and MGAV on serum cytokine profiles

**Fig 2:**
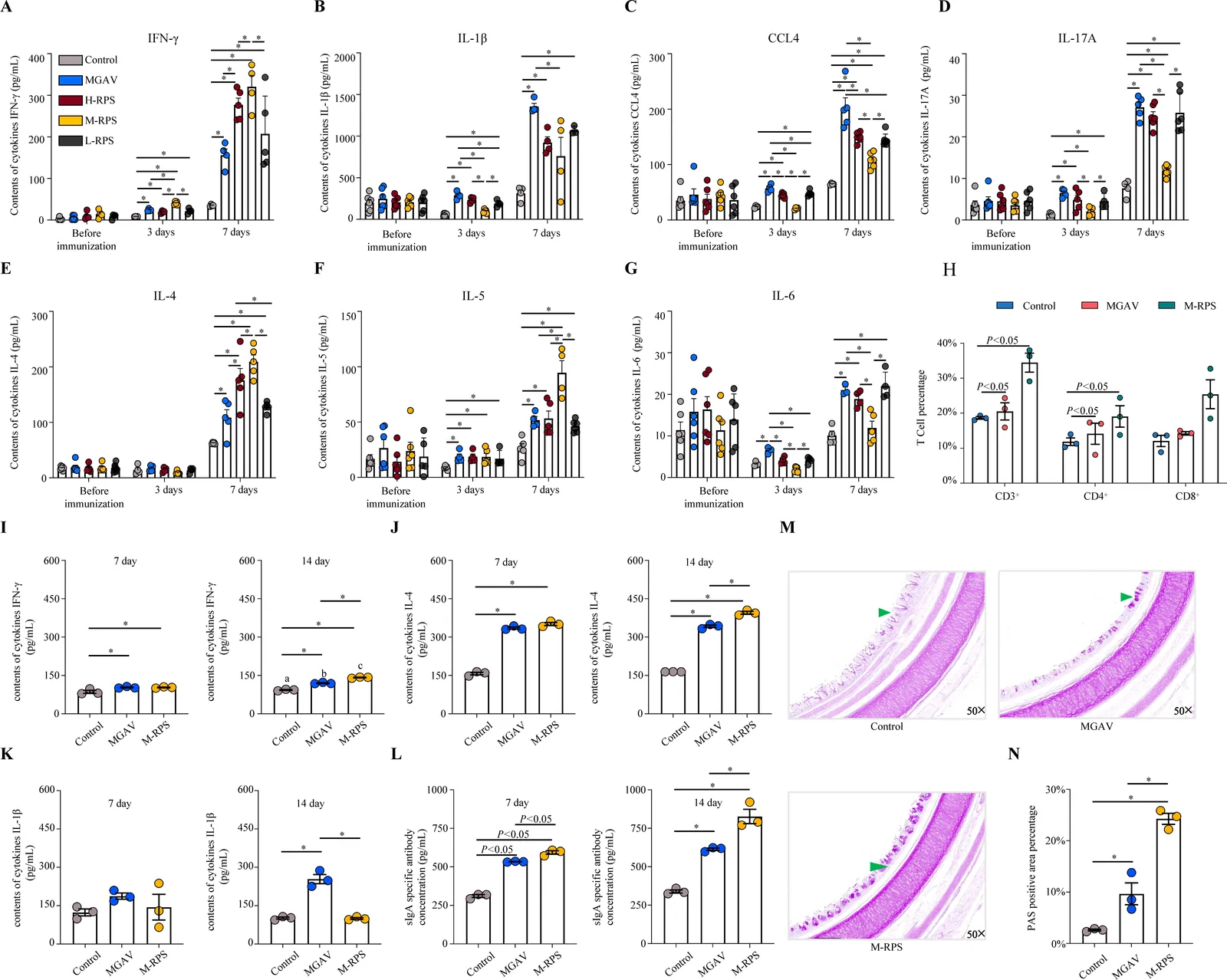
RPS enhances MGAV-induced serum cytokine production, T-cell responses, and mucosal barrier reinforcement in the trachea. (**A**) Serum IFN-γ levels. (**B**) Serum IL-4 levels. (**C**) Serum IL-5 levels. (**D**) Serum CCL4 levels. (**E**) Serum IL-6 levels. (**F**) Serum IL-1β levels. (**G**) Serum IL-17A levels. (**H**) Effect of RPS on MGAV-induced splenic T-cell immunity after intranasal co-administration. (**I**) Relative expression of IFN-γ in tracheal mucosa. (**J**) Relative expression of IL-4 in tracheal mucosa. (**K**) Relative expression of IL-1β in tracheal mucosa. (**L**) sIgA levels in tracheal mucus. (**M**) Representative images of PAS-stained tracheal sections (green triangles indicate PAS-positive goblet cells). (**N**) Quantitative analysis of PAS-positive area percentage. For panels** A –G**, serum samples were collected at the indicated time points post-primary immunization. For panels **I–L**, tracheal tissues and mucus were collected at 7 and 14 days post-primary immunization. Data are presented as mean ± SEM (*n* = 6). Serum samples were collected at the indicated time points post-primary immunization. Tracheal tissues and mucus were collected at 7 and 14 days post-primary immunization. Data are presented as the mean ± SEM (*n* = 6). Statistical significance is indicated by a horizontal line with an asterisk (* for *P* < 0.05). Non-significant (*P* > 0.05) pairwise comparisons are not marked.

To evaluate RPS-mediated immunomodulation in chickens, we measured the dynamic changes in serum cytokine levels post-immunization and found that intranasal co-administration of RPS with MGAV significantly altered serum cytokine profiles. Prior to immunization, no significant differences were observed in any cytokine across the groups. Following primary immunization, RPS enhanced Th1-associated cytokine levels: IFN-γ was significantly elevated in the M-RPS group at day 3, and by day 7, all three RPS groups showed increased IFN-γ, with M-RPS eliciting the strongest response (Fig. 2A). Similarly, the mRNA levels of IL-4 and IL-5 were significantly upregulated by day 7 in the H-RPS and M-RPS groups, respectively (Fig. 2B and C). In contrast, RPS administration suppressed multiple pro-inflammatory mediators. CCL4 was markedly reduced across all RPS doses at days 3 and 7, with the most pronounced decrease in the M-RPS group. IL-6 and IL-1β levels were significantly lower in all RPS-treated groups at day 3, and by day 7, suppression of IL-6 and IL-17A remained significant only in the M-RPS group, whereas IL-1β reduction persisted across all RPS doses (Fig. 2D through G). Collectively, these results demonstrated that RPS, particularly at 20 mg/kg, promotes a balanced Th1/Th2 activation while attenuating excessive inflammatory responses, highlighting its potential as an effective mucosal adjuvant for MGAV.

### Co-administered intranasal RPS and MGAV increases splenic T-lymphocyte subsets

To evaluate cellular immune responses in the control, MGAV, and M-RPS groups, the proportions of splenic T-lymphocyte subsets at 2 weeks post-primary immunization were analyzed by flow cytometry (Fig. 2H). Specifically, the frequencies of CD3^+^, CD4^+^, and CD8^+^ T cells in the M-RPS group were 34.46% ± 4.71%, 19.06% ± 5.33%, and 25.41% ± 7.14%, respectively. These frequencies were significantly higher than those in the control and MGAV groups, indicating that RPS, as a mucosal adjuvant, effectively enhances MGAV-induced T-cell immunity.

### Effects of RPS on the tracheal mucosa

Intranasal co-administration of RPS with MGAV significantly enhanced tracheal mucosal immunity, as evidenced by cytokine profiles and histological analysis. Compared with the MGAV group, the M-RPS showed no significant change in IFN-γ or IL-4 expression at 7 days post-primary immunization, but exhibited significant upregulation of both cytokines by day 14 (Fig. 2I and J). In contrast, IL-1β levels remained unchanged across all groups at day 7, but were significantly suppressed in the M-RPS group by day 14 (Fig. 2K). Furthermore, total sIgA levels in tracheal mucus were markedly elevated in the M-RPS group at both 7 and 14 days post-immunization compared with those in the MGAV group, with the most pronounced increase observed at the later time point (Fig. 2L). Histological examination of tracheal sections via PAS staining revealed varying degrees of purplish-red staining in mucosal goblet cells (Fig. 2M, green triangles). Quantitative analysis showed that both the MGAV and M-RPS groups exhibited a significant percentage increase in the PAS-positive area (reflecting goblet cell numbers) compared with the control group, with the M-RPS group demonstrating the most substantial expansion (Fig. 2N). Collectively, these findings indicated that RPS augments tracheal mucosal immunity by modulating cytokine expression, strengthening sIgA secretion, and promoting goblet cell hyperplasia.

### Protection against MG challenge

**Fig 3:**
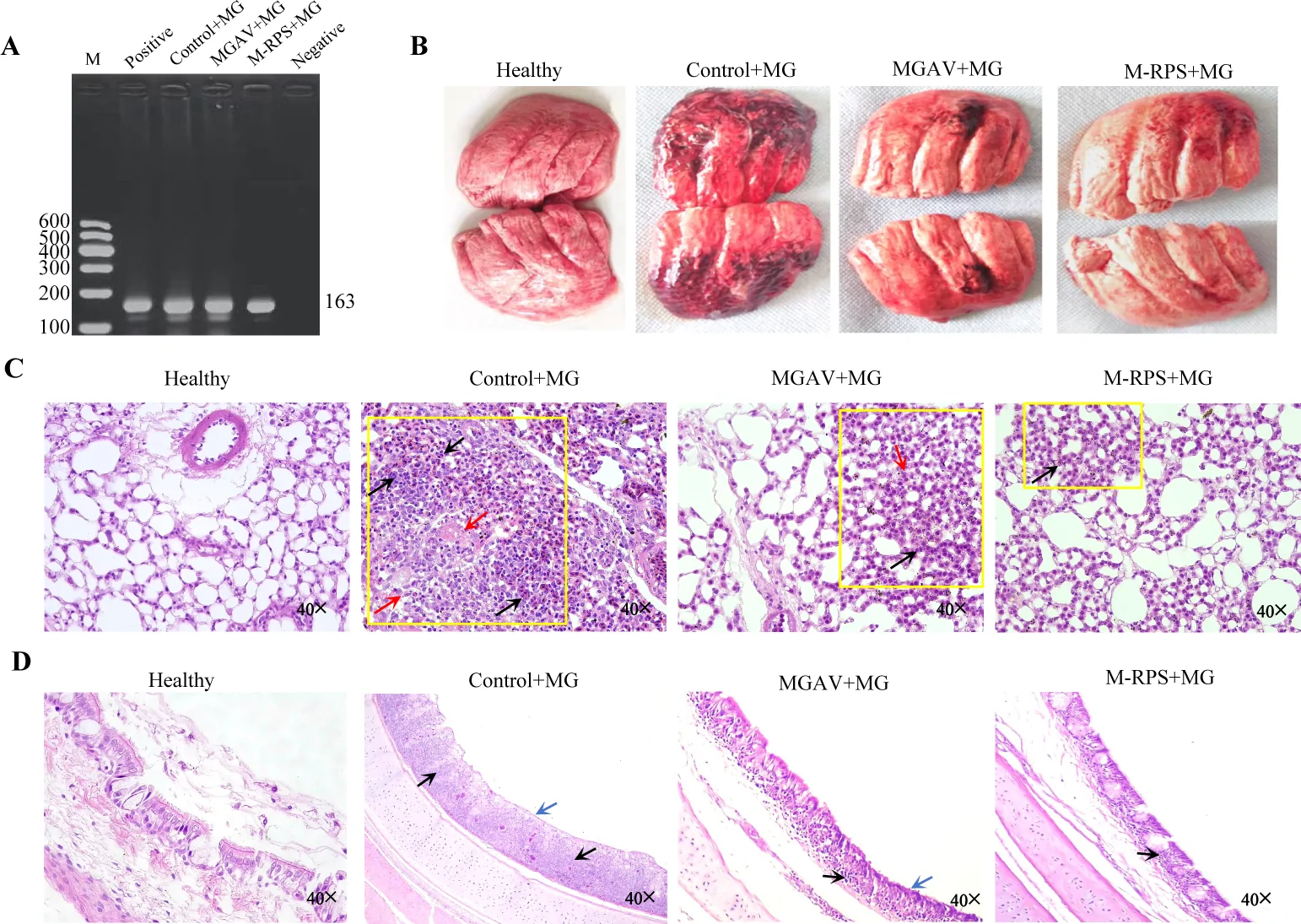
RPS enhances protection against MG challenge in immunized chickens. (**A**) Agarose gel electrophoresis of PCR products from lung tissues at 14 days post-challenge. (**B**) Macroscopic lung lesions and consolidation patterns. (**C**) H&E analysis of lung tissues; yellow boxes indicate areas of parenchymal pathology; black arrows show inflammatory cell infiltration with lymphoid follicle formation; and red arrows indicate epithelial hyperplasia and tissue consolidation. (**D**) H&E analysis of tracheal tissues; black arrows show inflammatory infiltration; and blue arrows indicate cilia loss.

At 14 days post-challenge with the MG mx-FZ2023 strain, PCR amplification of MG-specific DNA from lung tissues was confirmed by agarose gel electrophoresis, verifying successful infection (Fig. 3A). Macroscopic examination of the lungs showed varying degrees of consolidation across groups (Fig. 3B). Morbidity rates were 83.33% in the control group, 33.33% in the MGAV group, and 16.67% in the M-RPS group (Table 3). Corresponding average lung lesion areas were 65.4%, 5.8%, and 1.5%, with total lung lesion scores of 38, 5, and 2, respectively. Histopathological examination of lung (Fig. 3C) and tracheal (Fig. 3D) tissues via H&E staining further demonstrated the protective effect of RPS. Compared with the unchallenged healthy group, tracheal and lung lesions showed progressively decreased severity in the control, MGAV, and M-RPS groups. Lung sections from challenged birds exhibited varying degrees of parenchymal pathology (Fig. 3C, yellow boxes), including inflammatory cell infiltration often accompanied by lymphoid follicle formation (black arrows) and epithelial hyperplasia leading to tissue consolidation (red arrows). Tracheal sections showed loss of cilia (blue arrows) and severe inflammatory infiltration (black arrows) in all challenged groups. However, the M-RPS group displayed substantially milder mucosal damage in both lung and trachea compared with the other challenged groups. In contrast, the healthy group exhibited clear and intact pulmonary and tracheal structures without significant abnormalities. Collectively, these findings indicated that RPS significantly enhances the protective efficacy of MGAV, effectively reducing MG-induced pathological damage in the tracheal mucosa and lungs.

**TABLE 3:** Pulmonary lesion scores in challenged chickens 14 days post-infection^*a*^

Group	Healthy group		Control + MG group		MGAV + MG group		M-RPS + MG group	
	Lung lesion area (%)	Pulmonary lesion score	Lung lesion area (%)	Pulmonary lesion score	Lung lesion area (%)	Pulmonary lesion score	Lung lesion area (%)	Pulmonary lesion score
Left lung lesion rate 1	0	0	75	4	0	0	0	0
Right lung lesion rate 1	0	0	80	4	0	0	0	0
Left lung lesion rate 2	0	0	85	4	13	1	10	1
Right lung lesion rate 2	0	0	80	4	27	2	8	1
Left lung lesion rate 3	0	0	80	4	0	0	0	0
Right lung lesion rate 3	0	0	85	4	0	0	0	0
Left lung lesion rate 4	0	0	70	3	0	0	0	0
Right lung lesion rate 4	0	0	70	3	0	0	0	0
Left lung lesion rate 5	0	0	75	4	11	1	0	0
Right lung lesion rate 5	0	0	85	4	19	1	0	0
Left lung lesion rate 6	0	0	0	0	0	0	0	0
Right lung lesion rate 6	0	0	0	0	0	0	0	0
Mean ± SEM (area %)	0^a^		65.4 ± 13.2^b^		5.8 ± 3.7^a^		1.5 ± 1.5^a^	
Mean ± SEM (score)	0^a^		6.3 ± 1.3^b^		0.8 ± 0.5^a^		0.3 ± 0.3^a^	
Total score	0		38		5		2	

^*a*^ Individual values for each chicken are presented as left/right lung lesion area (%) and left/right pulmonary lesion score. For summary statistics, lung lesion area (%) was calculated as the average of the left and right lesion area percentages per bird, and the pulmonary lesion score was the sum of the left and right scores (0–8). Summary data are expressed as mean ± SEM. Different lowercase letters within a summary row indicate statistically significant differences among groups (*P* < 0.05). Groups sharing the same letter are not significantly different.

### Metagenomic insights into RPS-mediated modulation of the respiratory microbiota in MGAV-immunized chickens

**Fig 4:**
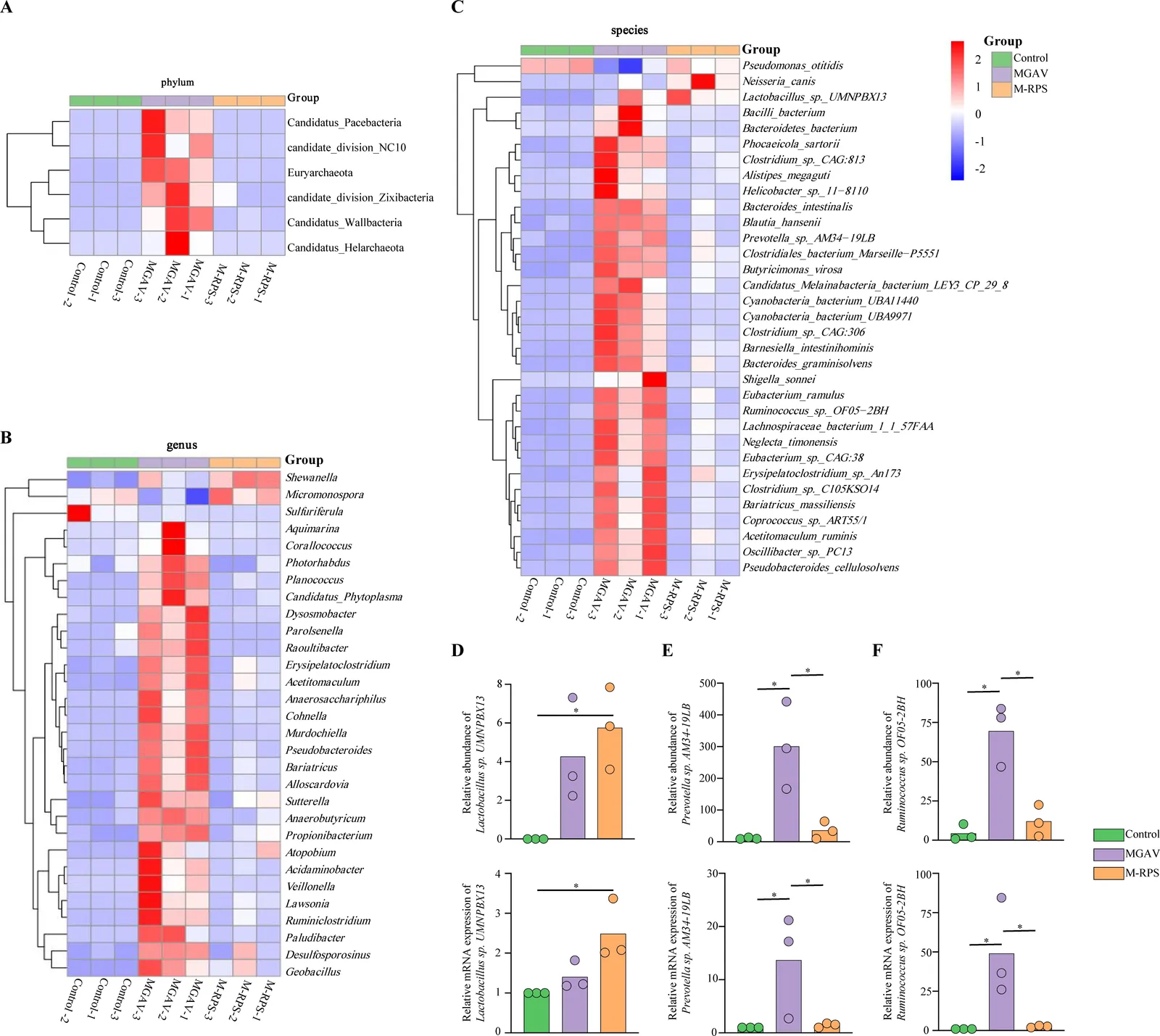
RPS modulates the respiratory microbiota composition in MGAV-immunized chickens. (**A**) Relative abundance of major bacterial phyla. (**B**) Heatmap showing relative abundance of differentially abundant genera. (**C**) Significantly altered bacterial species based on metagenomic sequencing. (**D–F**) qPCR validation of selected differential species: *Lactobacillus_sp._UMNPBX13* (**D**), *Prevotella_sp. _AM34-19LB* (**E**), and *Ruminococcus_sp._OF05-2BH* (**F**).

To investigate the impact of RPS on the respiratory microbiota, tracheal mucus samples were collected at 14 days post-primary immunization for metagenomic analysis. At the phylum level, RPS-treated groups showed significant reductions in the relative abundance of Firmicutes, Bacteroidetes, and Cyanobacteria compared with the control and MGAV groups (Fig. 4A). At the genus level, significant decreases were observed in three genera: *Lachnoclostridium*, *Ruminococcus*, and *Bacteroides* (Fig. 4B). Species-level analysis revealed that RPS significantly enriched beneficial species including *Pseudomonas otitidis*, *Neisseria canis*, *Lactobacillus* sp. UMNPBX13, and *Lactobacillus mucosae*, while it significantly suppressed the abundance of *Prevotella* sp. AM34-19LB, *Ruminococcus* sp. OF05-2BH, and *Clostridium* sp. CAG:306 (Fig. 4C). To validate the metagenomic findings, quantitative PCR (qPCR) was performed on selected differentially abundant bacterial species. The observed trends for *Lactobacillus* sp. UMNPBX13 (Fig. 4D), *Prevotella* sp. AM34-19LB (Fig. 4E), and *Ruminococcus* sp. OF05-2BH (Fig. 4F) were consistent with the sequencing data, confirming the reliability of the microbial community analysis.

### Metabolomic analysis reveals modulation of respiratory metabolites by co-administered RPS and MGAV

**Fig 5:**
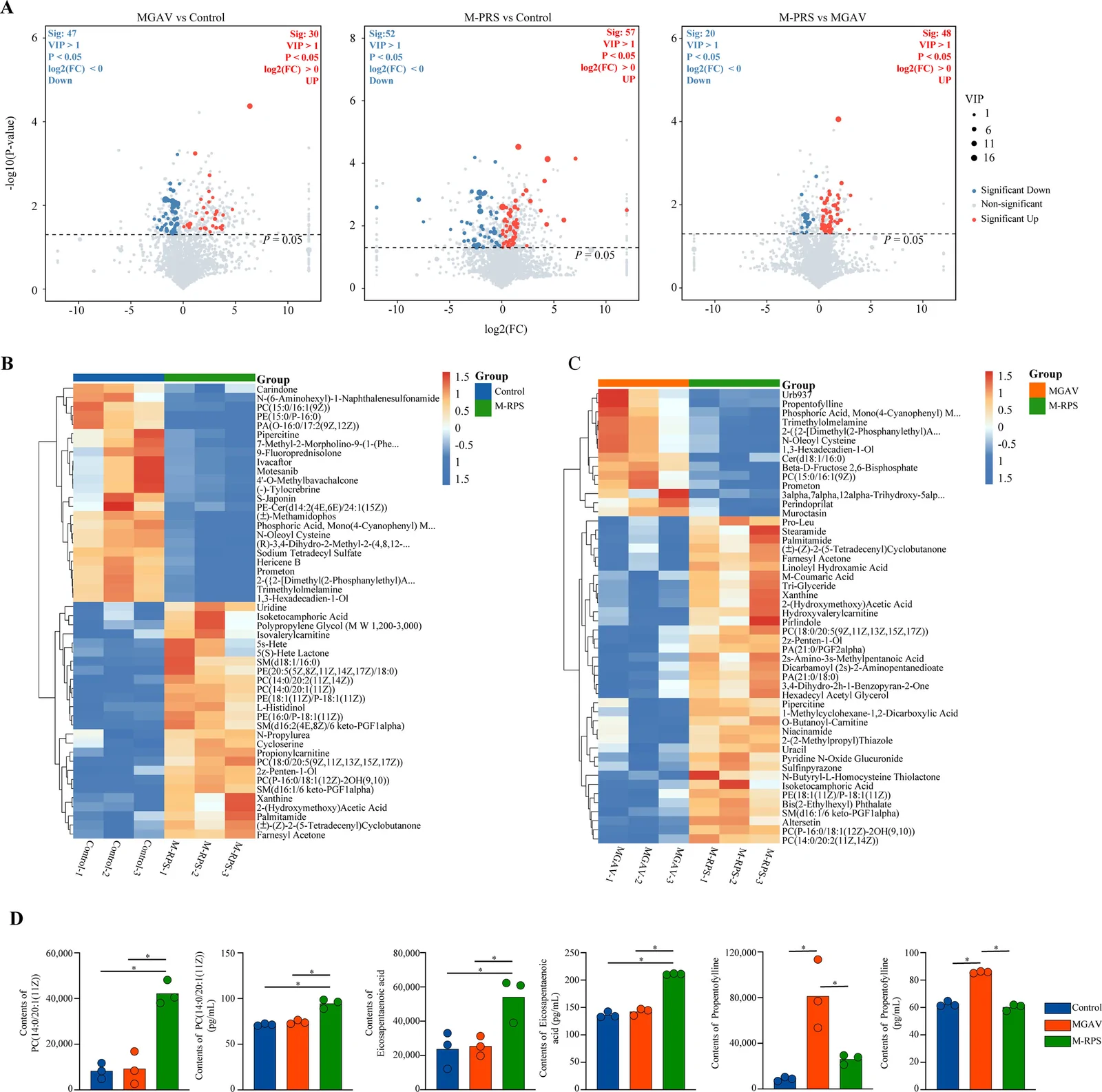
RPS combined with MGAV modulates the respiratory metabolomic profile in chickens. (**A**) Number of differentially abundant metabolites between experimental groups. (**B**) Heatmap of significantly altered metabolites and top 50 VIP-ranked metabolites in M-RPS vs control groups. (**C**) Heatmap of significantly altered metabolites and top 50 VIP-ranked metabolites in M-RPS vs MGAV groups. (**D–F**) ELISA validation of key metabolites: PC(14:0/20:2(11Z)) (**D**), eicosapentaenoic acid (**E**), and propentofylline (**F**).

Beneficial microorganisms enhance host immune function through the release or regulation of metabolites. To investigate alterations in respiratory metabolites following immunization, we performed metabolomic profiling of tracheal mucus samples. A total of 3,258 metabolites were detected across the control, MGAV, and M-RPS groups. Using criteria of VIP > 1 and *P* < 0.05, we identified 77, 109, and 68 differentially abundant metabolites in comparisons of MGAV vs control, M-RPS vs control, and M-RPS vs MGAV, respectively (Fig. 5A). Hierarchical clustering of significantly altered metabolites and the top 50 VIP-ranked metabolites revealed that, compared with the control group, the M-RPS group showed marked increases in 5(S)-hydroxyeicosatetraenoic acid (5S-HETE), l-histidinol, and PC (14:0/20:1[11Z]) (Fig. 5B). Relative to the MGAV group, the M-RPS group exhibited significant elevations in metabolites including PC (14:0/20:2[11Z]), phosphatidic acid (PA 21:0/PGF2α), phosphatidylethanolamine (PE 18:1[11Z]/P-18:1[11Z]), and sphingomyelin (SM d16:1/6 keto-PGF1α) (Fig. 5C). To confirm these findings, targeted ELISAs were conducted for three key metabolites: PC (14:0/20:2[11Z]), eicosapentaenoic acid, and propentofylline (Fig. 5D through F), revealing intergroup trends consistent with the metabolomics data and supporting the reliability of the identified metabolite profiles.

### Integrated metagenomic and metabolomic analysis reveals RPS-mediated microbiota–metabolite interactions

**Fig 6:**
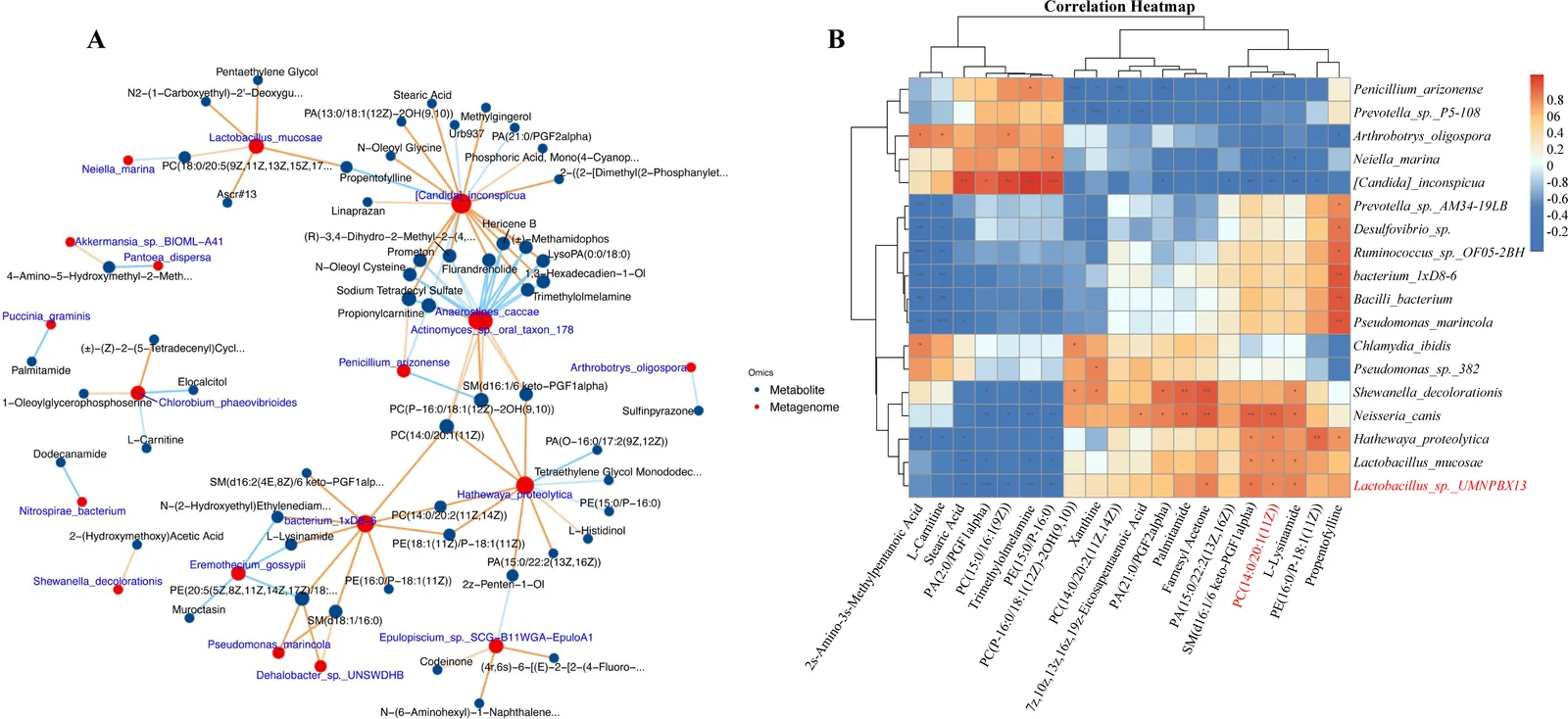
Integrated analysis reveals RPS-mediated microbiota-metabolite interactions in the respiratory tract. (**A**) Correlation network of the top 100 significant microbe-metabolite pairs. Bright yellow lines indicate positive correlations, while light blue lines indicate negative correlations; line thickness corresponds to the strength of correlation. Blue labels represent nodes from metagenomic data (microbial species), and black labels represent nodes from metabolomic data (metabolites). Node size indicates the degree of connectivity, and node color distinguishes the data type (microbe or metabolite) as shown in the legend. (**B**) Heatmap of Spearman correlation coefficients between differentially abundant microbial species and metabolites.

To elucidate the mechanism by which RPS modulates immunity, we performed an integrated analysis of metagenomic and metabolomic data. A correlation network was constructed using the top 100 microbe–metabolite pairs ranked by the absolute value of the correlation coefficient under significance criteria (Fig. 6A). This network revealed that *Lactobacillus* species enriched by RPS treatment, including *L.* sp. UMNPBX13 and *L. mucosae,* were positively correlated with several immunomodulatory metabolites, namely PC(14:0/20:2[11Z]), L-lysineamide, and SM(d16:1/6 keto-PGF1α). In contrast, bacterial taxa suppressed by RPS (e.g., *Prevotella* sp. AM34-19LB and *Ruminococcus* sp. OF05-2BH) were positively correlated with propentofylline and negatively correlated with metabolites including stearic acid and PA(2:0/PGF1α) (Fig. 6B). These findings suggested that RPS may enhance respiratory immunity by remodeling the microbial community, thereby altering the abundance of key metabolites involved in immune regulation.

### Effect of intranasal *Lactobacillus* administration on respiratory mucosal immunity

**Fig 7:**
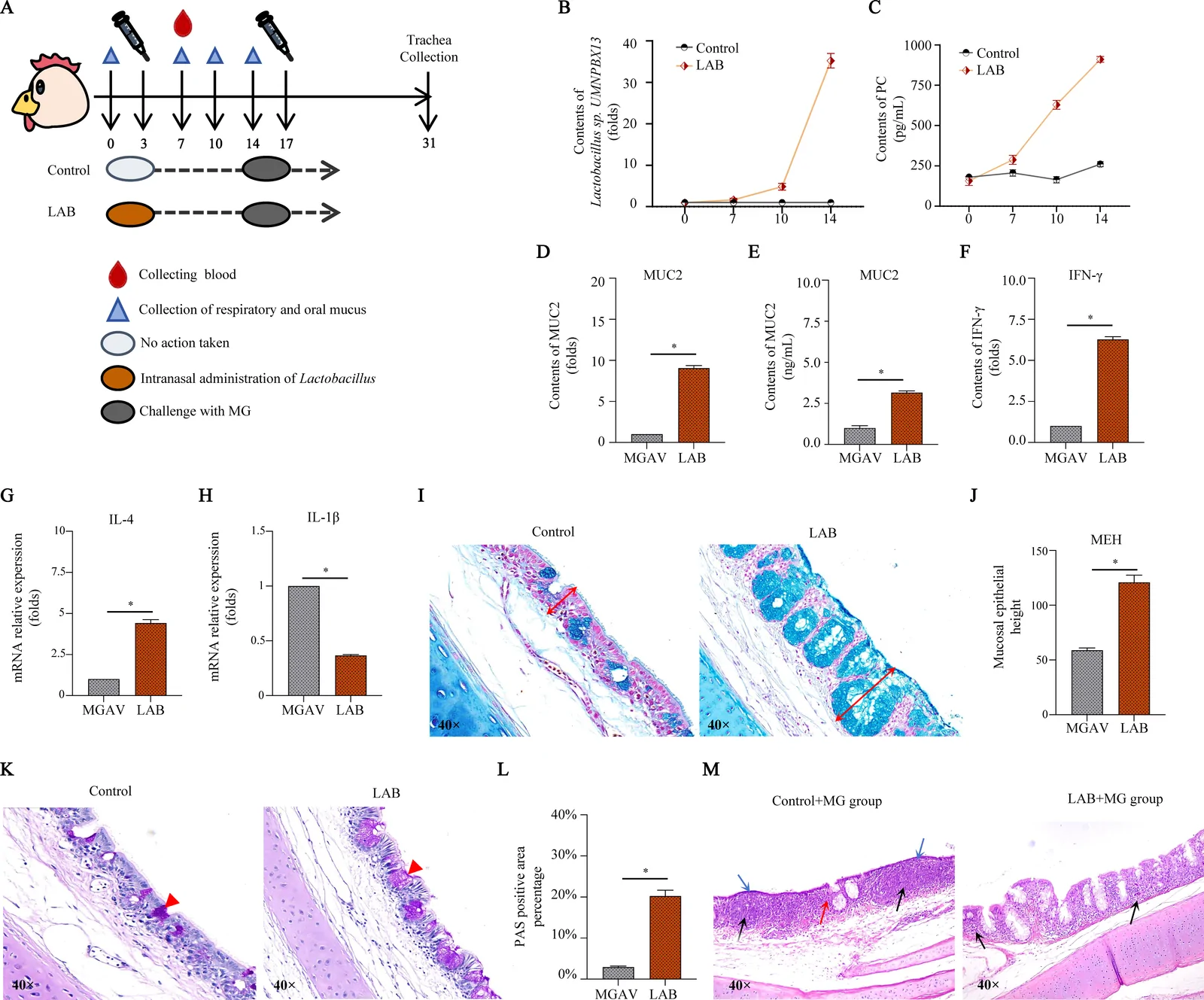
Intranasal administration of LAB enhances respiratory mucosal immunity and protects against MG challenge in chickens. (**A**) Relative abundance of LAB in the respiratory tract at 0, 7, and 14 days post-administration by RT-qPCR. (**B**) Levels of the metabolite PC measured by ELISA. (**C**) Relative expression of MUC2 mRNA by qPCR. (**D**) MUC2 protein levels by ELISA. (**E–G**) Serum cytokine levels: IFN-γ (**E**), IL-4 (**F**), and IL-1β (**G**) at 7 days post-administration. (**H and I**) Tracheal mucosal epithelial height shown by AB staining (**H**) and quantitative analysis (**I**). (**J and K**) Goblet cell response shown by PAS staining (**J**) and quantitative analysis of PAS-positive area (**K**). (**L**) Representative H&E-stained tracheal sections after MG challenge: blue arrows indicate ciliary loss, yellow arrows show reduced goblet cells, black arrows mark inflammatory cell infiltration. The results are presented as means ± SEM; *P < 0.05. Non-significant (*P* > 0.05) pairwise comparisons are not marked.

To investigate how intranasally delivered lactobacilli influence respiratory mucosal immunity, we assessed bacterial colonization, metabolite levels, and immune responses in chickens. The experimental design is schematically illustrated in Fig. 7A. RT-qPCR analysis revealed a continuous increase in the relative abundance of lactobacilli in the respiratory tract over 14 days following administration (Fig. 7B). Consistently, ELISA showed a concurrent rise in the metabolite PC (Fig. 7C). The expression of *MUC2*, a key mucin gene, was significantly upregulated in the LAB group, as confirmed by both qPCR and ELISA. These results reflected consistent trends across treatment groups (Fig. 7D and E). Systemic immune profiling at 7 days post-administration showed that, compared with the control group, serum levels of IFN-γ and IL-4 were significantly elevated (Fig. 7F and G), whereas IL-1β was significantly reduced (Fig. 7H), indicating a balanced Th1/Th2 response and attenuated inflammation in the LAB group. Histological examination further demonstrated the protective effect of lactobacilli on the tracheal mucosa. AB staining revealed a significant increase in tracheal mucosal epithelial height (Fig. 7I and J), while PAS staining showed a significant expansion in the PAS-positive area, reflecting goblet cell hyperplasia (Fig. 7K and L). To evaluate protection against infection, chickens were challenged with MG 14 days after lactobacillus administration. H&E staining of tracheal sections at two weeks post-challenge showed severe lesions in the control group, including ciliary loss (blue arrows), reduced goblet cells (yellow arrows), and marked inflammatory cell infiltration (black arrows). In contrast, the LAB group exhibited only mild inflammatory infiltration (Fig. 7M), indicating that lactobacillus pretreatment significantly alleviated MG-induced tracheal mucosal damage.

### PC enhances MGAV-acquired immunity by modulating cytokines and reinforcing mucosal defense

**Fig 8:**
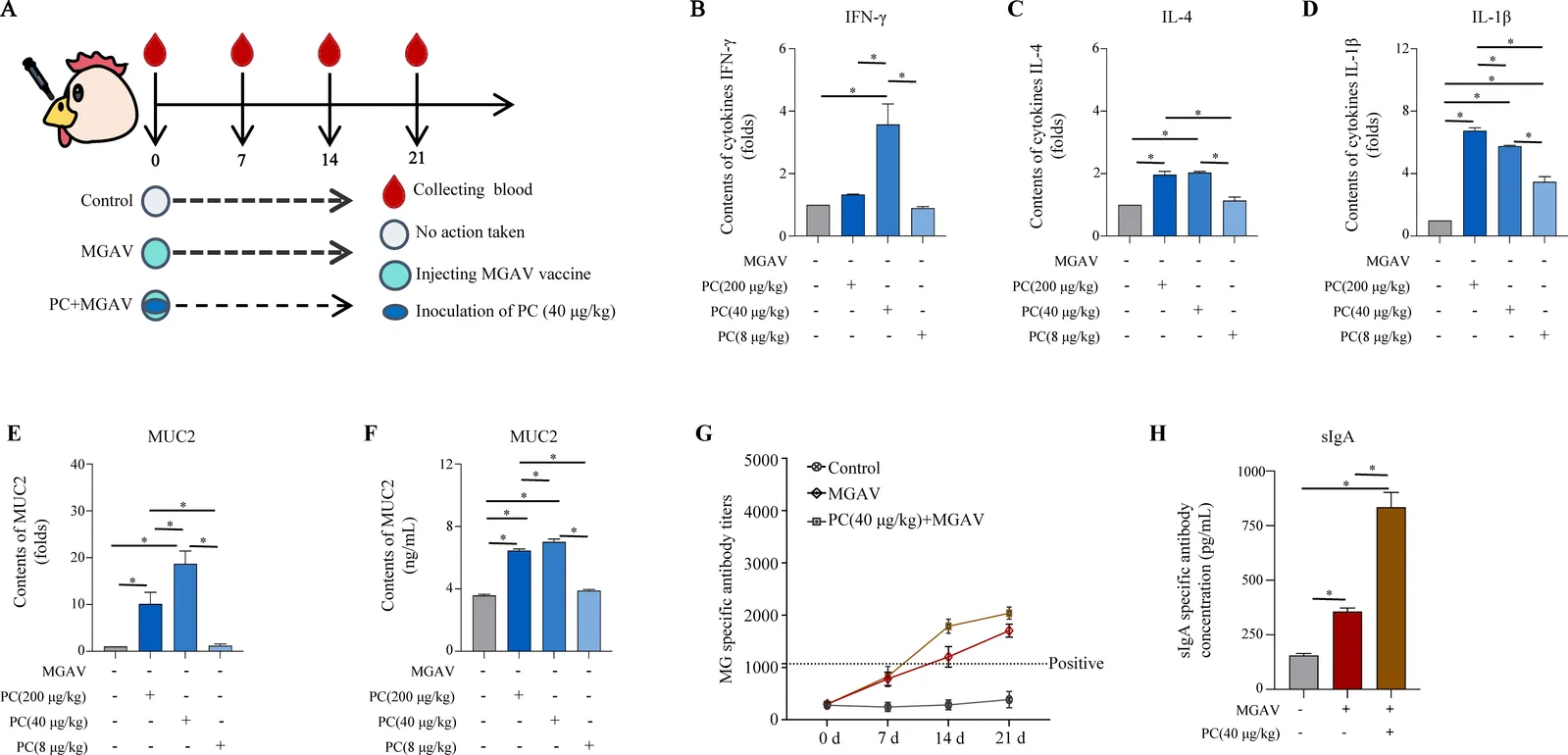
PC enhances MGAV-induced immunity by modulating cytokine responses and reinforcing mucosal defense in chickens. (**A**) Schematic diagram of the experimental timeline for evaluating the enhancement of MGAV-induced immune responses by PC. (**B–D**) Serum cytokine levels in chickens after intranasal PC administration: IFN-γ (**B**), IL-4 (**C**), and IL-1β (**D**). (**E**) Relative expression of MUC2 mRNA in tracheal mucosa. (**F**) MUC2 protein levels measured by ELISA. (**G**) MG-specific antibody titers post-immunization. (**H**) Total mucosal sIgA levels at 14 days post-immunization. For PC, *in vivo* doses were 200, 40, and 8 μg/kg, respectively. The results are presented as means ± SEM; *P < 0.05. Non-significant (*P* > 0.05) pairwise comparisons are not marked.

To investigate the effect of intranasal PC on MGAV-induced immunity, we conducted an experiment as schematically illustrated in Fig. 8A. In chickens receiving intranasal PC, only the 40 μg/kg PC group showed increased IFN-γ expression in whole blood relative to the control group at 7 days post-administration (Fig. 8B), whereas both the 8 and 200 μg/kg PC groups exhibited significantly elevated IL-4 levels (Fig. 8C). Furthermore, all three PC doses significantly enhanced IL-1β expression compared with the control, with the most pronounced increase observed at 40 μg/kg (Fig. 8D). In the tracheal mucosa, *MUC2* mRNA expression was markedly increased in both the 40 and 200 μg/kg PC groups, but not the 8 μg/kg group, relative to the control group (Fig. 8E). Consistently, ELISA results showed significant elevations in MUC2 protein levels in the 40 and 200 μg/kg PC groups, but not in the 8 μg/kg group (Fig. 8F). To determine whether PC enhances immune responses to MGAV, we measured MG-specific antibody levels. The control group showed no significant change, whereas significant increases were observed post-immunization in both the MGAV-alone and PC (40 μg/kg) + MGAV groups, with the most pronounced elevation in the PC (40 μg/kg) + MGAV group (Fig. 8G). Mucosal immunity exhibited a similar pattern: total sIgA levels at day 14 were significantly higher in the MGAV and PC (40 μg/kg) + MGAV groups than in the control group, with the combination treatment delivering the greatest increase (Fig. 8H).

## DISCUSSION

MG infection typically begins with colonization of the upper respiratory tract, followed by invasion of airway epithelial cells, triggering inflammation, excessive mucus production, and eventual systemic immune suppression (30). In this context, intranasal vaccination represents a rational strategy to induce mucosal immunity; however, it may also provoke localized inflammatory responses. Saponin-based adjuvants have been shown to enhance immunogenicity and modulate immune-related gene expression (31), yet their potential to balance immunopotentiation with inflammatory control remains underexplored. In the present study, we demonstrated that RPS, as a nasal adjuvant for MGAV, was well tolerated and promoted a more balanced immune response in the chicken respiratory tract. Specifically, RPS co-administration resulted in significantly reduced pro-inflammatory cytokine levels compared with MGAV alone, while enhancing the expression of key immunomodulatory cytokines and increasing the number of PAS-positive goblet cells. These findings suggest that RPS augments adaptive mucosal immunity while reinforcing the tracheal epithelial barrier, potentially mitigating inflammation-related tissue injury. In the present study, the observation that the M-RPS group (20 mg/kg) elicited higher MG-specific antibody titers than the H-RPS group (100 mg/kg) may be attributed to the possibility that the 20 mg/kg dose lies within the optimal adjuvant window, providing sufficient immunostimulant without overt toxicity, whereas the 100 mg/kg dose may have exceeded the mucosal tolerance threshold, causing local irritation or cellular damage that ultimately diminished the overall antibody response (23). Cytokine-mediated regulation is essential for maintaining immunological homeostasis, which depends on a balance between pro- and anti-inflammatory factors (32). In our study, RPS administration effectively promoted the expression of anti-inflammatory IL-4 while suppressing pro-inflammatory IL-1β. Following eosinophilic airway inflammation, CCL4 is initially released from airway epithelial cells and subsequently produced by activated eosinophils, lymphocytes, and macrophages, which accumulate in the airways in response to CCL4 signaling (33). In this study, both MGAV immunization and RPS treatment at all three doses were found to promote CCL4 production in serum. Notably, flavonoids from *Houttuynia cordata* effectively reduce the production of IL-8, tumor necrosis factor (TNF)-α, and monocyte chemoattractant protein-1 (also known as CCL2), while increasing IFN-β levels in the lungs, thereby breaking the cytokine–immune cell feedback loop via bidirectional immunomodulation. Organic acids (e.g., chlorogenic acid) and flavonoids from *Lonicera japonica* also suppress the production of IL-6, TNF-α, IL-1β, and reactive oxygen species in lipopolysaccharide-stimulated macrophages, inhibit IKKα/β and IκBα phosphorylation, and block nuclear factor-κB pathway activation, thereby preventing macrophage overactivation in acute inflammatory settings (34, 35). When MG invades its host, the respiratory mucosa serves as the primary site of infection. IgA is the predominant antibody at mucosal surfaces, and sIgA, its principal form, is recognized as the first line of immune defense against mucosal pathogens (36). By providing multiple antigen-binding sites, sIgA exhibits enhanced antigen affinity and potent neutralizing activity, thereby playing a critical role in local mucosal immunity (37). In this study, MGAV significantly elevated sIgA levels in the trachea, an effect that was further enhanced by co-administration with RPS.

Lactic acid bacteria, a predominant group of gut probiotics, contribute to intestinal microbial homeostasis through the production of lactic acid and other metabolites that suppress the growth of pathogenic bacteria (38). Analogously, commensal bacteria in the upper respiratory tract confer colonization resistance against potential pathogens, thereby lowering the incidence of respiratory infections. Even upon mucosal colonization by pathogens, commensals and their metabolites can restrict further microbial expansion and virulence (39). The respiratory mucosal mechanical barrier comprises diverse epithelial cells interconnected by tight junctions, among which goblet cells, ciliated cells, and basal cells serve essential protective functions. Specifically, goblet cells secrete mucins to form a mucus layer that traps and clears invaders, thereby safeguarding the underlying epithelium (40). Previous studies have indicated that beneficial bacteria, including *Lactobacillus casei* and *Lactococcus lactis*, can modulate pulmonary Th1/Th2 immune responses and stimulate the secretion of cytokines such as IL-4 and IFN-γ (41). In addition, lactobacilli have been shown to enhance pro-inflammatory cytokine production and strengthen overall host immunity, conferring protection against respiratory infections (42). Consistent with these findings, our study demonstrated that intranasally administered lactic acid bacteria significantly upregulated *MUC2* expression and PC levels in the respiratory mucosa, increased epithelial height and mucus volume, and elevated goblet cell numbers, collectively reinforcing the structural and functional integrity of the respiratory mucosal immune barrier.

PC serves as a structural substrate essential for maintaining the integrity of lipid bilayers in mucosal barriers, and its dietary intake has been shown to protect gastrointestinal mucosal function and suppress inflammatory activity (43). Our metabolomic analysis revealed that RPS treatment significantly increased PC levels in the chicken respiratory tract. Pulmonary surfactant is predominantly composed of phospholipids, including PC (44), and elevated PC levels in the lungs reflect turnover and epithelial proliferation of biomembranes (45). Previous work (46) indicated that PC alleviates endoplasmic reticulum stress and oxidative stress, inhibits Th17 cell differentiation, and promotes Treg differentiation. As core constituents of the pulmonary mucus in healthy airways, airway mucins are integral to the mucociliary defense system, thereby helping to shield the lungs from inhaled pathogens and environmental toxins (47). MUC2 is a high-molecular-weight glycoprotein produced by goblet cells. It forms a protective mucus barrier, thereby serving as a first line of innate host defense against pathogen invasion (48). PC has also been shown to bind MUC2 in the gut, forming a hydrophobic barrier that protects the intestinal epithelium from microbes and suppresses TNF-α-, IL-1β-, and IL-6-induced inflammatory responses (49). These findings suggest that PC plays an important role in maintaining barrier integrity and immune homeostasis, a conclusion further supported by our observation that PC modulates the expression of IFN-γ, IL-4, and IL-1β, thereby enhancing immune responses.

**Fig 9:**
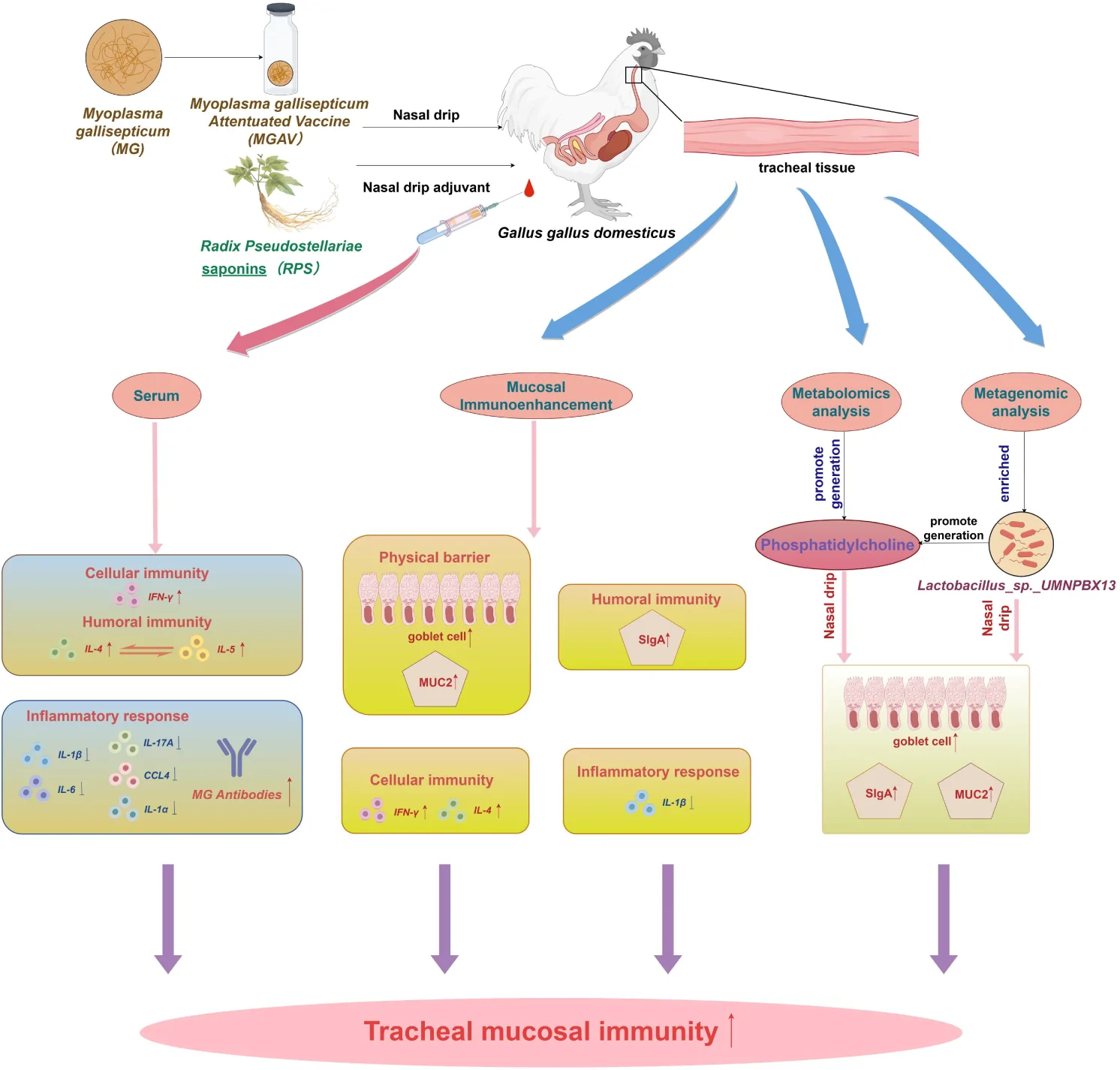
Scheme of RPS enhancing the immunity against *Mycoplasma gallisepticum* (by Figdraw).

In summary, this study highlights the ability of RPS to enhance mucosal immunity and potentially mitigate inflammatory injury by strengthening the local mucosal barrier, demonstrating considerable promise as an adjuvant for enhancing MGAV responses in chickens (Fig. 9). These findings not only offer a new strategy for poultry health protection but also open avenues for integrating traditional Chinese medicine-derived compounds into modern vaccine adjuvant development. Simultaneously, it should be noted that the present study focused exclusively on evaluating the protective effects of RPS against MG in a chicken model. Whether RPS exerts similar beneficial effects against other avian or mammalian pathogens, or in other animal species, remains to be determined.

### Conclusion

Our study identifies RPS as a promising mucosal immunomodulator that enhances respiratory immune function by optimizing the respiratory microbiota, particularly through increased abundance of lactic acid bacteria, which promote production of the metabolite PC. This cascade elevates MG-specific antibody levels and reinforces sIgA expression, thereby improving the immune response induced by MGAV. These findings highlight the potential of RPS to augment vaccine immunogenicity while offering mechanistic insight into its role in strengthening mucosal barrier function and mitigating inflammatory damage.

## Data Availability

Data will be made available on request, and the raw sequencing data from this experiment can be accessed under BioProject accession number PRJNA1078526.
